# Identifying Cancer genes by combining two-rounds RWR based on multiple biological data

**DOI:** 10.1186/s12859-019-3123-8

**Published:** 2019-11-25

**Authors:** Wenxiang Zhang, Xiujuan Lei (IEEE member), Chen Bian

**Affiliations:** School of Computer Science, Shaanxi Normal University, Xi’an, 710119 Shaanxi China

**Keywords:** Identify cancer genes, Quadruple layer heterogeneous network, Two-rounds random walk with restart, Multiple biological data

## Abstract

**Background:**

It’s a very urgent task to identify cancer genes that enables us to understand the mechanisms of biochemical processes at a biomolecular level and facilitates the development of bioinformatics. Although a large number of methods have been proposed to identify cancer genes at recent times, the biological data utilized by most of these methods is still quite less, which reflects an insufficient consideration of the relationship between genes and diseases from a variety of factors.

**Results:**

In this paper, we propose a two-rounds random walk algorithm to identify cancer genes based on multiple biological data (TRWR-MB), including protein-protein interaction (PPI) network, pathway network, microRNA similarity network, lncRNA similarity network, cancer similarity network and protein complexes. In the first-round random walk, all cancer nodes, cancer-related genes, cancer-related microRNAs and cancer-related lncRNAs, being associated with all the cancer, are used as seed nodes, and then a random walker walks on a quadruple layer heterogeneous network constructed by multiple biological data. The first-round random walk aims to select the top score k of potential cancer genes. Then in the second-round random walk, genes, microRNAs and lncRNAs, being associated with a certain special cancer in corresponding cancer class, are regarded as seed nodes, and then the walker walks on a new quadruple layer heterogeneous network constructed by lncRNAs, microRNAs, cancer and selected potential cancer genes. After the above walks finish, we combine the results of two-rounds RWR as ranking score for experimental analysis. As a result, a higher value of area under the receiver operating characteristic curve (AUC) is obtained. Besides, cases studies for identifying new cancer genes are performed in corresponding section.

**Conclusion:**

In summary, TRWR-MB integrates multiple biological data to identify cancer genes by analyzing the relationship between genes and cancer from a variety of biological molecular perspective.

## Background

A substantial amount of diseases is generally triggered by single or multiple mutations and associated with one or more genes [1]. The diseases associated with multiple genes will be classified as polygenic disorders (complex disorders), such as Alzheimer disease, cancer disease, obesity disease and so on [2]. Compared to Mendelian disorders, the prevalence of complex diseases is higher, and for complex diseases, the genetic model is more complicated because they violate Mendel’s laws of inheritance and involve in more pathogenic genes [3]. This kind of diseases accounts for more than 80% of human diseases and seriously threatens human health, but the mechanism of emergence and development is still unclear [4–6]. Therefore, the identification of complex disease genes has become an urgent and sophisticated task in the field of bioinformatics.

In recent years, with the rapid development of gene chip and high-throughput sequencing technology, multiple biological data, such as protein-protein interaction data [7], protein complexes data [8], pathway data [9–11], microRNA data [12] and lncRNA data [13], are growing exponentially. It provides a new perspective to explore the mechanism of emergence and development of complex diseases. However, a large number of algorithms, such as the logistic regression [14], the Bayesian method [15], direct neighbours of biological network [16], have a common drawback: they rarely analyze associations between diseases and genes out of multiple biological perspectives to predict disease genes and uncover the mechanism of complex diseases. In other words, these methods integrate fewer biological data in the course of identification of disease genes.

In order to make up for this defect, many new methods have been proposed based on these multiple biological data. Chen et al. [17] extracted corresponding features based on multiple biological data to identify disease genes by a logistic regression algorithm. Yang et al. [18] integrated four human weighted gene networks and constructed a new much larger weighted biological network to identify disease genes based on information entropy.

Although these aforementioned methods could be well used in analyzing associations between diseases and genes from the perspective of various biological networks, they merely considered the direct neighbours of the candidate genes in the corresponding networks, and ignored the fact that two genes as non-neighbors in some biological networks still have some biological relationships. Recently, some methods based on topological similarity have been proposed to solve this problem. Kohler S et al. [19] proposed a random walk with restart (*RWR*) and diffusion kernel methods to capture global topological relationship in an interactive network. In identifying disease genes, *RWR* achieved an outstanding performance compared to previous methods. Based on *RWR*, Li et al. [20] proposed an extension of *RWR,* which applied *RWR* to heterogeneous networks (*RWRH*). What followed was that a lot of improved algorithms were proposed based on *RWR* and *RWRH*. Luo et al. [21] applied an improved *RWR* algorithm to reconstruct PPI network, and ran *RWRH* on heterogeneous network constructed by integrating the reconstructed PPI network and disease similarity network, to identify disease genes. Li et al. [22] proposed a random walk on multigraphs merging heterogeneous genomic and phenotype data (*RWRM*), which can capture multiple edges between a pair of nodes to identify disease-associated genes. Valdeolivas et al. [23] extended the random walk with restart on multiplex and heterogeneous biological networks, which is beneficial to explore different layers of functional and physical interactions between genes and proteins. However, these methods still have a shortcoming: the less seed nodes during the process of walking. It will give occasion to weak ability to mine potential disease genes.

In this study, we propose an extension of *RWRH* algorithm to explore a quadruple layer heterogeneous network, which is constructed by combining PPI network, pathway network, microRNA similarity network, lncRNA similarity network, cancer similarity network, and protein complexes. There are three highlights of *TRWR-M*B: (1) Constructing a quadruple layer heterogeneous network; (2) Two-round random walk with restart on the quadruple layer heterogeneous network; (3) Aggregate the results of two-round random walk with restart into a final ranking score. In these three, the core of our study is two-round random walk with restart. In the first-round random walk with restart, we can select highly suspicious candidate genes, which embrace highly probability of being related to all cancer. In the second-round random walk with restart, we can select highly suspicious candidate cancer genes that are associated with a special cancer from the results of the first-round of random walk. Next, after the two rounds of random walk finish, we combine two results to obtain the final ranking score. Finally, we apply the *TRWR-MB* algorithm to predict new candidate genes in breast cancer, lung cancer, colon cancer, prostate cancer and leukemia.

## Methods

In this section, we describe the TRWR-MB algorithm in details. Firstly, we will introduce the motivation of TRWR-MB. Next, we will introduce the corresponding materials, the construction of a quadruple layer heterogeneous network, and two-round random walk with restart, respectively. Finally, a flowchart of TRWR-MB will be presented.

### Motivations

As described in the previous section, TRWR-MB has three highlights. A quadruple layer heterogeneous network is constructed by fusing PPI network, pathway network, microRNA similarity network, lncRNA similarity network, cancer similarity network and protein complexes; TRWR-MB identifies cancer genes in the quadruple layer heterogeneous network; A final ranking score is calculated by combining the results of two-round random walk with restart. Next, we will introduce our motivations for the three highlights.

Recently, a lot of methods [19–23] have been proposed based on network topological similarity. However, these methods have two shortcomings. Firstly, seed nodes are too less in the process of random walk with restart. Secondly, these methods only use gene or protein interactive network. Besides, none of the aforementioned methods has considered the effects of lncRNA and microRNA on identifying disease genes. Therefore, we construct a quadruple heterogeneous network by using genes, proteins, microRNAs, lncRNAs and cancer. Moreover, protein complexes are used as a positive feedback to enhance cancer-related interaction. The quadruple heterogeneous network not only considers the effects of lncRNAs and microRNAs on cancer, but also can increase the number of seed nodes based on cancer-related lncRNAs and microRNAs in the process of random walk with restart.

Marc et al. [24] suggested that the similarity among phenotypes are positively correlated with a number of measures of gene function, including relatedness at the level of protein sequence, protein motifs, functional annotation, and direct protein-protein interaction. Therefore, we hypothesize that similar diseases have a great probability of being linked to the same genes. Goh et al. [1] manually classified multiple diseases into cancer class based on the physiological system. Being inspired by [25, 26], we propose a two-round random walk with restart to identify cancer genes. In the first-round of random walk with restart, our purpose aims to select highly suspicious candidate genes related to cancer class (all cancer diseases) and remove majority of noise genes. In the second-round of random walk with restart, our purpose is to select cancer genes from the highly suspicious genes selected in the first-round of random walk with restart. In the end, a final ranking score is calculated by balancing the results in cancer class and special cancer.

### Materials

#### Biological network

The datasets of PPI network are downloaded from the Human Protein Reference Database (HPRD) (Release 9) [7]. The HPRD database contains protein interaction data in the file of HPRD_Release9_041310.tar.gz, where we can link two human genes if their corresponding protein interacts with each other. We first map protein into the Entrez gene code, and then delete repeating protein-protein entries and each protein interacting with itself. The final PPI network consists of 9519 nodes and 37,048 edges.

The pathway datasets are obtained from the database of KEGG [10], Reactome [9], PharmGKB [11]. The pathway network is constructed by R packages graphite based on the aforementioned pathway database. The final pathway network consists of 10,717 nodes and 302,546 edges.

Because PPI network and pathway network embrace their own bias and relevance, we merge them to construct a gene network,which follows Li et al. [22]. Finally, the gene network consists of 13,596 nodes and 331,127 edges (deleting repeat edges).

#### Cancer-Cancer similarity network

Firstly, we extract cancer class, which contains a lot of cancer phenotypes, from Goh et al. [1], and then get cancer phenotype OMIM id [27]. Next, we extract Entrez terms of genes, which are associated with the corresponding cancer phenotypes, from the morbidmap.txt of OMIM database (being downloaded in Dec-2017). Finally, cancer class embraces 76 cancer phenotypes, 160 cancer genes that belong to the nodes of gene network, and 251 gene-cancer associations.

For cancer-cancer similarity network, many previous methods have been proposed [20–23]. We calculate cancer similarity by employing Valdeolivas et al. [23] methods, which use the relevance of the shared phenotypes to calculate disease similarity based on Phenotype Ontology Project (HPO) database [28]. The cancer-cancer similarity network is constructed by linking every cancer to its three nearest cancers according to cancer similarity. The number of interactions is 155 in cancer-cancer similarity network.

#### MicroRNA-cancer association and microRNA functional similarity network

In this paper, human microRNA-disease associations are downloaded from HMDD v3.0 database [12]. We delete some microRNA-disease entries, in which the disease doesn’t have corresponding OMIM id. Besides, the duplicated associations between the same microRNAs and diseases are also deleted. Finally, the dataset of microRNA-disease, which is used to construct microRNA similarity network, contains 310 diseases (having corresponding OMIM id), 940 microRNAs and 9454 microRNA-disease associations. The dataset of microRNA-cancer, which is constructed by deleting some microRNA-disease associations in the dataset of microRNA-disease in which diseases do not belong to cancer category, contains 38 cancer diseases, 810 microRNAs and 4297 microRNA-cancer associations.

In the process of constructing microRNA functional similarity network, we firstly calculate the similarity among 310 diseases, which is same with the computation of cancer similarity. Next, we estimate the functional similarity between two microRNAs as mentioned [28, 29], which can be computed as follows:

1 $$ \mathrm{D} Sim\left(d,D\right)=\underset{1\le i\le k}{\max}\left( DSim\left(d,{d}_i\right)\right) $$

2 $$ {\displaystyle \begin{array}{l}\mathrm{MiSim}\left({\mathrm{MiRNA}}_1,{\mathrm{MiRNA}}_2\right)=\\ {}\kern2.75em \frac{\sum \limits_{1\le i\le m} DSim\left({d}_{1i},{D}_2\right)+\sum \limits_{1\le j\le n} DSim\left({d}_{2j},{D}_1\right)}{m+n}\end{array}} $$

where *DSim*(*d,d*_*i*_) represents the similarity between a special disease d and a disease *d*_*i*_, which is the same as the method of calculating the similarity among cancers. *DSim*(*d,D*) is the greatest similarity score between a disease d and a disease group D. Besides, *d*_*nk*_ represents the disease k associated with *MiRNA*_*n*_. Similarly, *D*_*n*_ represents the disease group n, in which all diseases are associated with *MiRNA*_*n*_. *MiSim*(*MiRNA*_1_, *MiRNA*_2_) is the similarity score between *MiRNA*_1_ and *MiRNA*_2_.

Finally, we calculate all similarities among microRNAs to construct microRNA similarity matrix, and then we construct microRNA function similarity network by linking each microRNA to its 10 nearest neighbors according to microRNA similarity matrix. The microRNA function similarity network consists of 940 microRNAs and 8385 edges.

#### LncRNA-cancer association and lncRNA similarity network

We first download the known lncRNA-disease associations from the data_v2017.xls of LncRNADisease database [13]. However, the disease names are not standard, because we only find disease names but can’t find a standard index (e.g. OMIM id, DOID etc.) in the LncRNADdisease database. Therefore, we list all disease names first and then manually match them with the DOID based on Disease Ontology (DO) [30]. Besides, those diseases, which cannot be matched to the DOID, will be deleted, and each corresponding DOID of them has been listed in Additional file 1. Next, the lncRNA-disease associations consist of 188 diseases, 700 lncRNAs and 1344 lncRNA-disease associations.

Next, the functional similarity between two lncRNAs is measured, which can be computed as follows:

3 $$ \mathrm{D} Sim\left(d,D\right)=\underset{1\le i\le k}{\max}\left( DSim\left(d,{d}_i\right)\right) $$

4 $$ {\displaystyle \begin{array}{l}\mathrm{LncSim}\left({\mathrm{LncRNA}}_1,{\mathrm{LncRNA}}_2\right)=\\ {}\kern2.75em \frac{\sum \limits_{1\le i\le m} DSim\left({d}_{1i},{D}_2\right)+\sum \limits_{1\le j\le n} DSim\left({d}_{2j},{D}_1\right)}{m+n}\end{array}} $$

where *DSim*(*d,d*_*i*_) represents the similarity between a special disease d and a disease *d*_*i*_. It is calculated by the DOSE package of R based on DOID. The definition of *DSim*(*d,D*), *d*_*nk*_, *D*_*n*_ and *LncSim*(*LncRNA*_1_, *LncRNA*_2_) is similar to the corresponding definition in the last subsection. Similarly, we also construct lncRNA similarity matrix by *LncSim*(*LncRNA*_1_, *LncRNA*_2_), and link each lncRNAs to its ten nearest neighbours according to the lncRNA similarity matrix to construct lncRNA similarity network, which consists of 700 lncRNAs and 5349 edges.

Besides, because the disease names are not standard, we also manually match them with the OMIM id of cancer based on OMIM database. The lncRNA-cancer associations consist of 347 lncRNAs, 40 cancers and 839 lncRNA-cancer associations.

#### MicroRNA-gene interaction

MicroRNA-gene interaction data is downloaded in the database of miRTarBase [31]. Here, we just download the supported interactions for reliability. Finally, we extract 736 microRNAs and 2566 target genes, which are contained in the gene network and microRNA functional similarity network. The number of microRNA-gene interactions is 8046.

#### MicroRNA-lncRNA interaction

The microRNA-lncRNA associations can be downloaded in the database of starBase v2.0 [32]. In order to get a reliable interactive network, we only download the microRNA-lncRNA associations consisting of 5217 microRNA-lncRNA interactions about 274 microRNAs and 554 lncRNAs when the number of supporting experiments is greater than 1 or equal to 1. Besides, we delete some microRNA-lncRNA interactions, in which microRNAs and lncRNAs are not in the microRNA similarity network and lncRNA similarity network, respectively. Finally, the dataset consists of 45 microRNAs, 31 lncRNAs and 146 microRNA-lncRNA interactions.

#### LncRNA-gene interaction

LncRNA-gene interactions are downloaded in the database of NPInter [33], which collect 491,416 interactions of ncRNA with other biomolecules from 22 organisms. We only collect the interactions between the lncRNAs from lncRNA similarity network and the genes from the gene network. Finally, the data consists of 207 lncRNAs, 114 genes and 1122 lncRNA-gene interactions.

#### Protein complexes

Human protein complexes are downloaded from the database of CORUM [8]. After deleting protein complexes with a single protein, there are 3169 proteins and 2298 protein complexes.

#### Statistics of materials

The details of the data are shown in Table 1.

**Table 1:** Detail information of the data

Description	Value
Number of nodes in PPI network	9519
Number of interactions in PPI network	37,048
Number of nodes in pathway network	10,717
Number of interactions in pathway network	302,546
Number of nodes in gene network	13,596
Number of interactions in gene network	331,127
Number of protein complexes	2298
Number of proteins in protein complexes	3169
Number of nodes in cancer-cancer similarity network	76
Number of interactions in cancer-cancer similarity network	155
Number of genes associated with cancer	160
Number of gene-cancer associations	251
Number of nodes in microRNA functional similarity network	940
Number of edges in microRNA functional similarity network	8385
Number of microRNA in microRNA-gene interactions	736
Number of genes in microRNA-gene interactions	2566
Number of microRNA-gene interactions	8046
Number of microRNA in microRNA-cancer associations	810
Number of cancers in microRNA-cancer associations	38
Number of microRNA-cancer associations	4297
Number of nodes in lncRNA functional similarity network	700
Number of edges in lncRNA functional similarity network	5349
Number of lncRNA in lncRNA-gene interactions	207
Number of genes in lncRNA-gene interactions	114
Number of lncRNA-gene interactions	1122
Number of lncRNA in lncRNA-cancer associations	347
Number of cancers in lncRNA-cancer associations	40
Number of lncRNA-cancer associations	839
Number of lncRNA in microRNA-lncRNA interactions	31
Number of microRNA in microRNA-lncRNA interactions	45
Number of microRNA-lncRNA interactions	146

### Constructing a quadruple layer heterogeneous network

In order to increase the seed nodes of the random walk and consider the lncRNAs and microRNAs’ effects on cancer, we construct a quadruple layer heterogeneous network based on genes (or proteins), microRNAs, lncRNAs, cancer and the interactions among them.

In this paper, we suppose *G*_*n×n*_, *M*_*m×m*_, *L*_*l×l*_, *C*_*c×c*_, *GM*_*n×m*_, *GL*_*n×l*_, *GC*_*n×c*_, *ML*_*m×l*_, *MC*_*m×c*_ and *LC*_*l×c*_ are adjacency matrixes of gene network, microRNA similarity network, lncRNA similarity network, cancer-cancer similarity network, gene-microRNA interactions, gene-lncRNA interactions, gene-cancer associations, microRNA-lncRNA functional similarity network, microRNA-cancer associations and lncRNA-cancer associations, respectively. And n, m, l and c represent the number of genes, microRNAs, lncRNAs and cancer, respectively. The adjacency matrix of the quadruple layer heterogeneous network can be represented as follows:

5 $$ \mathrm{H}=\left[\begin{array}{cccc}{G}_{n\times n}& \mathrm{G}{M}_{n\times m}& {GL}_{n\times l}& {GC}_{n\times c}\\ {}\mathrm{G}{M}_{n\times m}^T& {M}_{m\times m}& {ML}_{m\times l}& {MC}_{m\times c}\\ {}{GL}_{n\times l}^T& {ML}_{m\times l}^T& {L}_{l\times l}& {LC}_{l\times c}\\ {}{GC}_{n\times c}^T& {MC}_{m\times c}^T& {LC}_{l\times c}^T& {C}_{c\times c}\end{array}\right] $$

where $\mathrm{G}{M}_{n\times m}^T$, ${GL}_{n\times l}^T$, ${ML}_{m\times l}^T$, ${GC}_{n\times c}^T$, ${MC}_{m\times c}^T$ and ${LC}_{l\times c}^T$ are the transposes of G*M*_*n* × *m*_, *GL*_*n* × *l*_, *GC*_*n* × *c*_, *ML*_*m* × *l*_, *MC*_*m* × *c*_ and *LC*_*l* × *c*_, respectively.

### Calculating transition matrix

Subsequently, *W*, the transition matrix, need to be constructed for random walks based on the adjacency matrix of *H* as follows:

6 $$ \mathrm{W}=\left[\begin{array}{cccc}\left(1-\delta \right){W}_G& \frac{\delta }{3}{W}_{\mathrm{G}M}& \frac{\delta }{3}{W}_{GL}& \frac{\delta }{3}{W}_{GC}\\ {}\frac{\delta }{3}{W}_{GM^T}& \left(1-\delta \right){W}_{\mathrm{M}}& \frac{\delta }{3}{W}_{ML}& \frac{\delta }{3}{W}_{M\mathrm{C}}\\ {}\frac{\delta }{3}{W}_{GL^T}& \frac{\delta }{3}{W}_{ML^T}& \left(1-\delta \right){W}_L& \frac{\delta }{3}{W}_{LC}\\ {}\frac{\delta }{3}{W}_{GC^T}& \frac{\delta }{3}{W}_{MC^T}& \frac{\delta }{3}{W}_{LC^T}& \left(1-\delta \right){W}_C\end{array}\right] $$

where *W*_M_, *W*_*L*_, *W*_*C*_, *W*_G*M*_, *W*_*GL*_, *W*_*GC*_, *W*_*ML*_, *W*_*M*C_ and *W*_*LC*_ are the row-normalizing matrixes of M_m × *m*_, L_*l* × *l*_, C_c × *c*_, GM_*n* × *m*_, G*L*_*n* × *l*_, *GC*_*n* × *c*_, ML_m × *l*_, MC_*m* × *c*_ and L*C*_*l* × *c*_. What’s more, ${W}_{GM^T}$, ${W}_{GL^T}$, ${W}_{ML^T}$, ${W}_{GC^T}$, ${W}_{MC^T}$ and ${W}_{LC^T}$ have similar definitions. Besides, *δ* ∈ [0, 1] controls the probability of staying in the same layer network or jumping to different layer network for random walkers.

The single biological network usually contains a lot of noises. Therefore, adding some other biological data, such as protein complexes, is helpful for identifying disease-related genes [17]. Because of this, we combine PPI network and pathway network to construct a transition matrix of multigraphs merging biological network, which is inspired by [22]. Besides, protein complexes are used to analyze cancer genes from a functional perspective of proteins.

Firstly, we construct a gene network by combining PPI and pathway network. The transition matrix of PPI can be defined as follows:

7 $$ {\mathrm{W}}_P\left(i,j\right)=\left\{\begin{array}{c}P\left(i,j\right)/{d}_P(i)\\ {}0\end{array}\begin{array}{c},\\ {},\end{array}\right.{\displaystyle \begin{array}{c}\kern1em if\kern0.5em P\left(i,j\right)\ne 0\&{d}_P(i)\ne 0\\ {}\mathrm{otherwise}\end{array}} $$

where *P* is the adjacency matrix of PPI network. *d*_*P*_(*i*) is the sum of i-th row for *P*. The definition of transition matrix of pathway network W_*Path*_ is similar to W_*P*_ ‘s. Then, we determine whether the node is a discrete point in the corresponding network as follows:

8 $$ {N}_p(i)=\left\{\begin{array}{c}1\\ {}0\end{array}\right.{\displaystyle \begin{array}{c},\\ {},\end{array}}{\displaystyle \begin{array}{c}\kern0.5em if\kern0.5em {d}_p(i)>0\\ {} otherwise\end{array}} $$

9 $$ {\mathrm{N}}_{Path}(i)=\left\{\begin{array}{c}1\\ {}0\end{array}\right.{\displaystyle \begin{array}{c},\\ {},\end{array}}{\displaystyle \begin{array}{c}\kern0.5em if\kern0.5em {d}_{Path}(i)>0\\ {} otherwise\end{array}} $$

10 $$ \mathrm{N}={N}_P+{N}_{\mathrm{path}} $$

obviously, the value of *N* can only take 1 or 2. If the value is equal to 1, it represents that the corresponding node only interacts with other nodes in one network. If the value is equal to 2, it represents that the corresponding node has interactions with other nodes in PPI network and pathway network.

In order to add a positive feedback to enhance cancer-related interaction, we consider protein complexes when the transition matrix of gene network is constructed, as follows:

11 $$ {\mathrm{W}}_{com}\left(i,j\right)=\left\{\begin{array}{c}{Num}_{com\_ dg}/{\mathrm{Num}}_{com\_g}\\ {}0\end{array}\right.{\displaystyle \begin{array}{c},\\ {},\end{array}}{\displaystyle \begin{array}{c}\kern0.5em if\kern0.5em G\left(i,j\right)>0\\ {} otherewise\end{array}} $$

where we suppose gene *i* is in a special protein complex. *Num*_*com* _ *dg*_ and Num_*com* _ *g*_ represent the number of cancer proteins (genes) and protein (genes) in the corresponding special protein complexes, respectively. If gene (protein) *i* is in multiple protein complexes, we select the maximum value of *Num*_*com* _ *dg*_/Num_*com* _ *g*_ as W_*com*_(*i*, *j*). The definition of *Initial_WG* obeys the follows rules:

12 $$ {\displaystyle \begin{array}{l}\mathrm{Initial}\_{\mathrm{W}}_G={\left[1/N\kern0.5em \cdots \kern0.5em 1/N\right]}_{\mathrm{n}\times n}\ast {W}_P+\\ {}\kern6em {\left[1/N\kern0.5em \cdots \kern0.5em 1/N\right]}_{n\times n}\ast {W}_{P\mathrm{ath}}+{W}_{com}\end{array}} $$

where A ∗ B belongs to Hadamard (elementwise) product. *W*_*G*_ is equal to the row-normalizing matrix of Initial _ W_*G*_.

### Two-round random walk with restart

#### The first-round random walk with restart

As mentioned in motivation section, the boundary among similar diseases caused by a set of functional similar genes is blurred. Therefore, we set all cancer-related genes, cancer-related microRNAs, cancer-related lncRNAs and cancer as seed nodes in the first step random walk with restart. Its purpose is to select a set of functional similar genes for cancer disease. After the first-round random walk with restart is done, the top *k* of genes score is selected as the set of functional similar genes, which are used to reconstruct a new quadruple heterogeneous network for the second-round random walk with restart. Here, we make k = *σn*, (*σ* ∈ [0, 1]), where *n* represents the number of genes.

In the first-round RWR, the initial probability vector can be denoted as:

13 $$ \mathrm{P}(0)=\eta \ast \left[\begin{array}{c}{\mathrm{g}}_0\\ {}\begin{array}{l}{m}_0\\ {}{\mathrm{l}}_0\end{array}\\ {}{c}_0\end{array}\right] $$

where the vector parameter $\eta =\left[{\eta}_1\kern0.5em {\eta}_2\kern0.5em {\eta}_3\kern0.5em {\eta}_4\right],\left({\eta}_{\mathrm{i}}\in \left[0,1\right]\right)$ is used to measure the importance of every layer network, and the sum of *η* is equal to 1. g_0_, *m*_0_, l_0_ and *c*_0_ denote the initial probability vector of gene network, microRNA similarity network, lncRNA similarity network and cancer-cancer similarity network, respectively. Then the random walk with restart is performed according to as follows:

14 $$ \mathrm{P}\left(t+1\right)=\left(1-\gamma \right) WP(t)+\gamma P(0) $$

where *γ* ∈ [0, 1] is the restart probability of walker in every walking. After some iterations, the P(∞) will enter a stable state when $\sqrt{{\left(\mathrm{P}\left(t+1\right)-P(t)\right)}^2}$ is less than 10^− 6^.

#### The second-round random walk with restart

We can get a set of functional similar genes after the first-round step random walk with restart. Then, we reconstruct a new quadruple layer heterogeneous network, and the second-round random walk with restart is employed in the new layer heterogeneous network to identify a special cancer gene.

The difference with the first random walk is that the seeds are selected from the special cancer nodes, cancer-related genes, cancer-related microRNAs and cancer-related lncRNAs, which are associated with the corresponding cancer. Other equations are similar with the first-round random walk with restart.

#### Getting the final score by combining the results of two-round random walks

As the theory mentioned above, the boundary among similar diseases is very vague, and it is not comprehensive that only consider the results of the second-round random walk. In our proposed method, the results of two-rounds of random walk are combined, which follow the rules below.

15 $$ \mathrm{Score}=\alpha {\mathrm{P}}^1\left(\infty \right)+\left(1-\alpha \right){P}^2\left(\infty \right) $$

where P^1^(∞) and *P*^2^(∞) represent the final results of first-round random walk with restart and second-round random walk with restart, respectively. The range of *α* is from 0 to 1, and *α* can adjust the importance of P^1^(∞) and *P*^2^(∞) in the final score.

#### A general framework

**Fig. 1:**
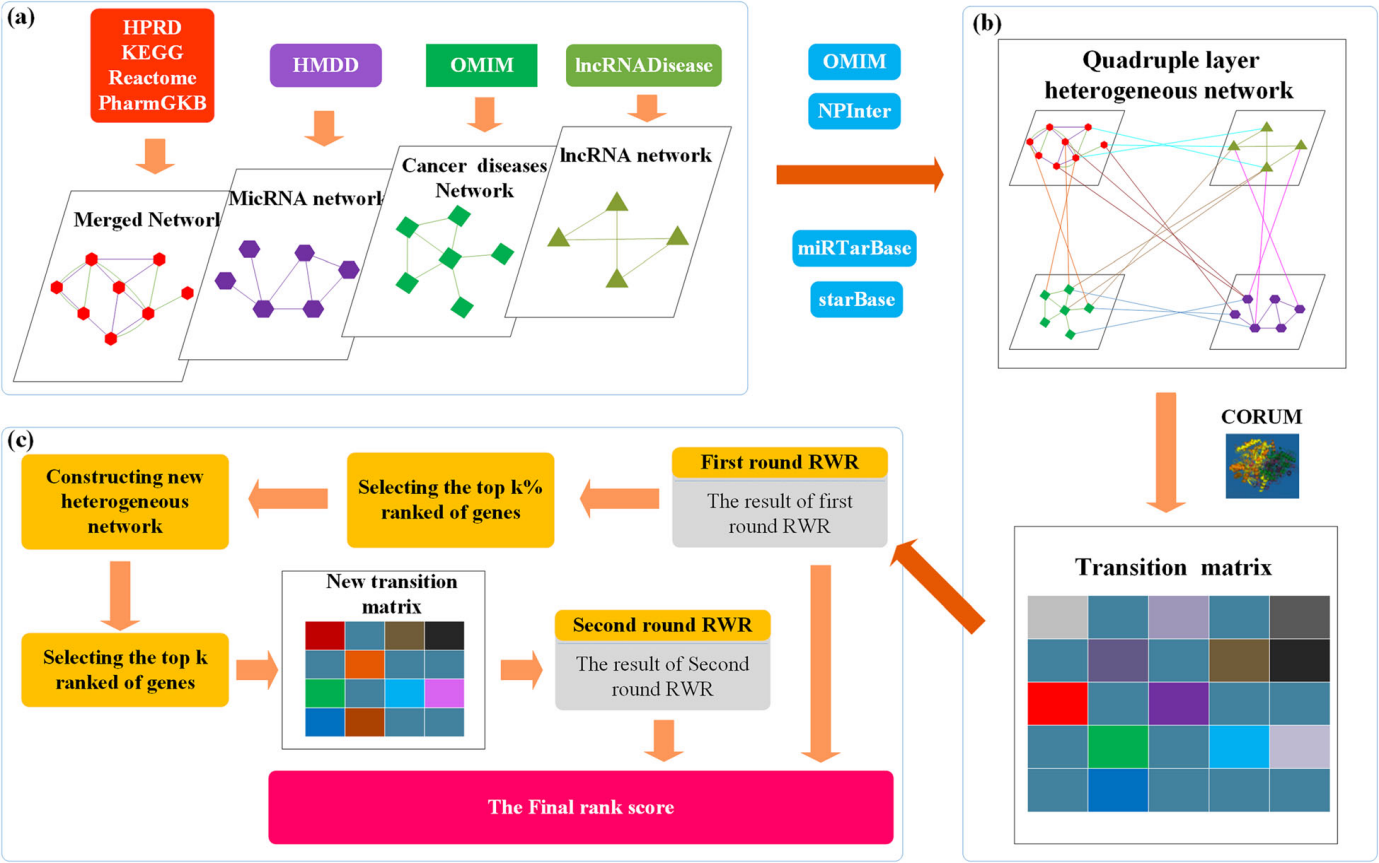
The framework of TRWR-MB: **a** showing data processing, which contains constructing merged network, microRNA network, cancer disease network, and lncRNA network. **b** constructing a quadruple layer heterogeneous network based on (**a**), and calculating the transition matrix based on the quadruple layer heterogeneous network and protein complexes. **c** identifying cancer-related gene by TRWR-MB

In this subsection, an overall framework for TRWR-MB is shown in Fig. 1.

## Results

### Evaluation criteria

After the two-round random walk, we obtain the Score and use it directly in experimental analysis. Inspired by [17], we employ the ranking score method to avoid the Score in different distributions for different diseases. For a gene *i*, the ranking score can be calculated as follows:

16 $$ Rank\_ score(i)=\frac{count\left( Score(i)> Score(j)\right)}{n},\left(i\ne j\right) $$

where *count*(*Score*(*i*) > *Score*(*j*)) represents how many times the *ith* gene’s *Score*(*i*) is greater than the *jth* gene’s *Score*(*j*). Obviously, a larger probability for gene *i* indicates that the gene *i* has a higher probability of being related to corresponding disease.

In this paper, we apply the leave-one-out across validation (LOOCV) to validate the performance of the algorithm. For each cancer disease, each known gene is left out in turn, and all the genes without relation to this specific cancer disease are placed in the candidate genes set. Our ultimate purpose is that the gene left out get a higher-ranking score than other genes in candidate genes set for a special cancer disease. Besides, the positive samples are known genes associated with cancer disease, and negative samples are genes without association with all cancer diseases.

According to the result of LOOCV, the ROC curve is presented by plotting the true positive rate (*TPR*) against the false positive rate (*FPR*) at various threshold settings. *TPR* and FPR are defined as follows:

17 $$ \mathrm{TPR}=\frac{\mathrm{TP}}{TP+ FN} $$

18 $$ \mathrm{FPR}=\frac{\mathrm{FP}}{TN+ FP} $$

where *TP* is the true positive, *TN* is the true negative, *FN* is false negative, and *FP* is the false positive. The area under the curve (AUC) value is computed based on ROC curve.

In the process of LOOCV, the number of the genes left out in the top *k%* of Rank_score is also a good evaluation criterion for the identification of cancer genes.

### The effects of parameters

In our algorithm, there are five parameters. Among them, we set *δ* = 0.5, *η*_i_ = 0.25 based on previous studies [23]. However, the value of *γ*, *σ* and *α* is undefined. Therefore, we make *γ* ∈ [0.1,   0.9], *σ* ∈ [0.2,  0.9] and *α* ∈ [0, 1] with an increment of 0.1.

**Fig. 2:**
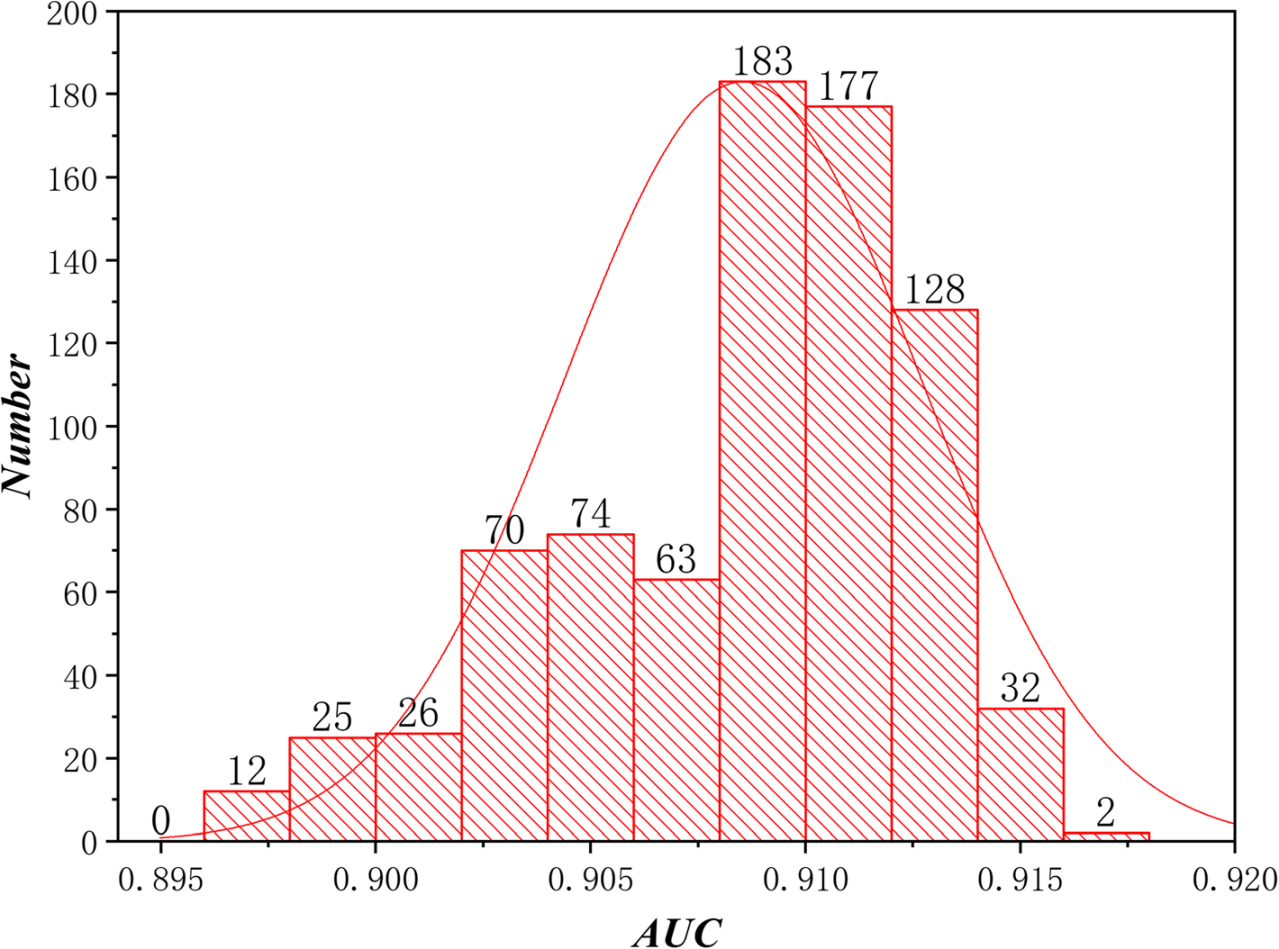
The histogram of AUC for all results

All detail results are put in Additional file 2. Besides, in Table 2, we put *δ* = 0.5, *η*_i_ = 0.25, *σ* = 0.6 and *γ* ∈ [0.1,   0.9], *α* ∈ [0, 1] with an increment of 0.1 and shows that we get the best result when *α* = 0.9. Besides, we plot the distribution of all AUC values showed in Fig. 2. Obviously, it can be clearly seen from Fig. 2 that the all AUC values obey the normal distribution, which can prove the scientificity of our algorithm.

**Table 2:** The AUC result for *α* ∈ [0, 1] with an increment of 0.1

	α	0	0.1	0.2	0.3	0.4	0.5	0.6	0.7	0.8	0.9	1
γ	AUC											
0.1		0.8961	0.8996	0.9013	0.9024	0.9027	0.9037	0.9035	0.9036	0.9034	0.9028	0.9032
0.2		0.9004	0.9045	0.9060	0.9070	0.9082	0.9087	0.9088	0.9086	0.9086	0.9084	0.9078
0.3		0.9027	0.9068	0.9086	0.9094	0.9099	0.9104	0.9111	0.9115	0.9112	0.9105	0.9086
0.4		0.9039	0.9082	0.9098	0.9103	0.9110	0.9117	0.9121	0.9128	0.9126	0.9117	0.9083
0.5		0.9047	0.9088	0.9104	0.9111	0.9115	0.9120	0.9125	0.9124	0.9132	0.9127	0.9075
0.6		0.9061	0.91020	0.9118	0.9132	0.9136	0.9148	0.9152	0.9158	0.9170	**0.9178**	0.9138
0.7		0.9055	0.9098	0.9110	0.9119	0.9125	0.9130	0.9136	0.9140	0.9149	0.9160	0.9109
0.8		0.9054	0.9089	0.9100	0.9112	0.9114	0.9121	0.9126	0.9133	0.9139	0.9148	0.9090
0.9		0.9047	0.9082	0.9090	0.9097	0.9103	0.9110	0.9115	0.9116	0.9126	0.9134	0.9073

*δ* = 0.5, *η*_i_ = 0.25, *σ* = 0.6, 0.9178 is bold, which represent the best of auc value

### Comparison with other algorithms

At the same time, we compare TRWR-MB (*δ* = 0.5, *η*_i_ = 0.25, *σ* = 0.6, *γ* = 0.6 and *α* = 0.9) with other four methods based on the topology of network, which are random walk with restart (RWR) [19], random walk with restart on heterogeneous network (RWRH) [20], random walk with restart on multigraphs merging heterogeneous (RWRM) [22], and random walk with restart on multiplex and heterogeneous Biological Networks (RWRMH) [23].

**Fig. 3:**
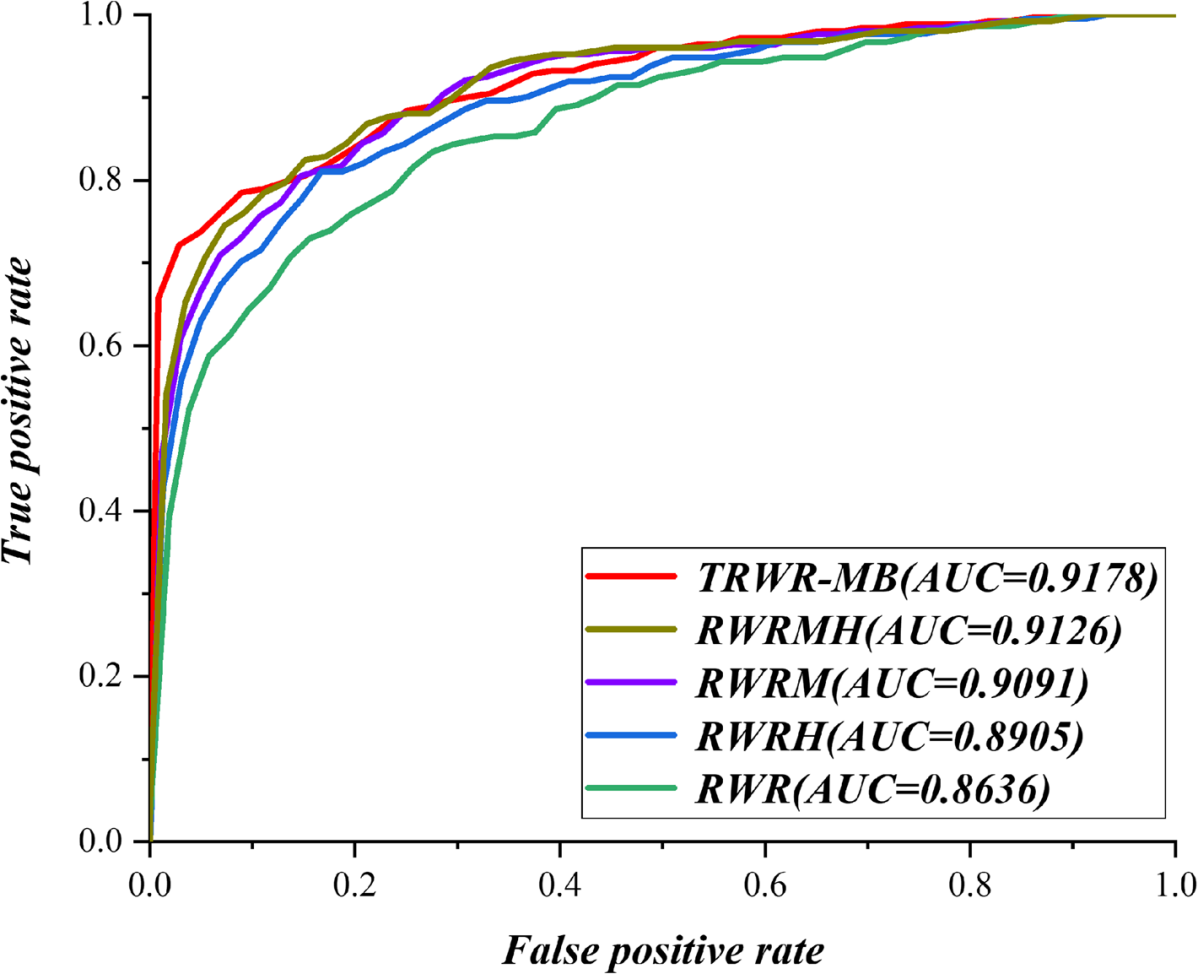
Comparison between TRWR-MB and other algorithms

The comparison results are shown in Table 3 and Fig. 3. We can observe from Table 3 that TRWR-MB get the best performance in each situation with different top *k%* of *Rank_score*. Figure 3 shows the ROC curve and the AUC value of TRWR-MB and other algorithms. Obviously, we can see that TRWR-MB performs best.

**Table 3:** The number of cancer genes in the top k% of Rank_score

Algorithms	TOP 5%	TOP 7%	TOP 10%	TOP 15%
TRWR-MB	**23**	**28**	**31**	**47**
RWRMH	20	24	**31**	43
RWRM	21	23	28	42
RWRH	20	20	27	41
RWR	18	22	28	38

where k is equal to 5, 7, 10 and 15, respectively; The five position in bold represent the best reseult

### Case study

To further validate the effectiveness of TRWR-MB (*δ* = 0.5, *η*_i_ = 0.25, *σ* = 0.6, *γ* = 0.6 and *α* = 0.9) for prioritizing new cancer disease genes, we list the top 10 candidate genes for 5 multifactorial cancer diseases to perform case studies here. The results are showed in Table 4. The cancer-related genes, cancer-related microRNAs, cancer-related lncRNAs, and corresponding cancer disease are used as the seed nodes. We only select Breast cancer (MIM: 114480) from these five to analyze for TRWR-MB, and only give other cancer-related genes PMID. The result shows that the effectiveness of TRWR-MB for identifying candidate cancer-related genes.

**Table 4:** The prediction result of new cancer genes

Rank	Breast cancer (MIM:114480)		Lung cancer (MIM:211980)		Colon Cancer (MIM:114500)		Prostate cancer (MIM:176807)		Leukemia (MIM:601626)	
	Gene	PMID	Gene	PMID	Gene	PMID	Gene	PMID	Gene	PMID
1	BRCA1	25,329,591	TP53	27,182,622	STK11	–	TP53	27,375,016	PDGFRB	29,133,777
2	NF1	–	EXT1	30,032,850	MLH1	28,224,663	RNASEL	–	BCR	–
3	PTEN	28,844,858	BLM	–	FH	–	HSPA1A	–	NF1	–
4	AXIN2	26,514,524	PIK3R1	–	NFKBIB	–	FGFR3	–	PTPN11	27,859,216
5	PLAG1	–	MAPK12	–	MSH2	28,537,674	MAD2L1	–	CBL	28,082,680
6	FOXO1	28,397,066	PIK3C2A	–	OAZ1	–	CTNNB1	29,229,583	ARHGAP26	–
7	GPC3	–	PIK3C2B	–	PIK3R1	–	EGFR	27,793,843	IL12RB2	–
8	WT1	29,016,617	RAF1	28,884,046	HRAS	–	STK11	–	MAPK12	–
9	CAV1	25,945,613	NF1	24,535,670	KRAS	27,338,794	MYC	–	TP53	27,959,731
10	DICER1	26,460,550	CNKSR1	–	GSK3B	–	MAX	29,108,267	DOT1L	27,294,782

If the cancer-related genes aren’t verified by literature, the correspond PMIDs are marked as -

Breast cancer is a kind of cancer which develops from breast tissue. Bilateral involvement and familial occurrence are important genetic factors. As shown in Table 4, the first prediction of breast cancer is BRCA1, which is a tumor suppressor involved in basic cellular functions necessary for cell replication and DNA synthesis, and Romagnolo et al. [34] indicated the natural food components that hold potential preventive effect against those types of breast cancer in which BRCA1 expression is either reduced or lacking. The second prediction of PTEN was confirmed to be the target of miR-221/222 in breast cancer cells [35]. Aristizabalpachon AF et al. [36] demonstrated that disturbance of β-catenin destruction complex expression and the defects of AXIN2 might be found in breast cancer patients. For the prediction of FOXO1, Liu et al. [37] provided an evidence that miR-9 can enhance the proliferation, migration, and invasion of breast cancer cells through down-regulating FOXO1. Xie et al. [38] revealed that breast cancer metastasis is affected by miR-193a-WT1 interaction. Shi et al. [39] suggested that human breast cancer cells and tissues can be observed to enhanced autophagy level and downregulation of CAV1.

**Fig. 4:**
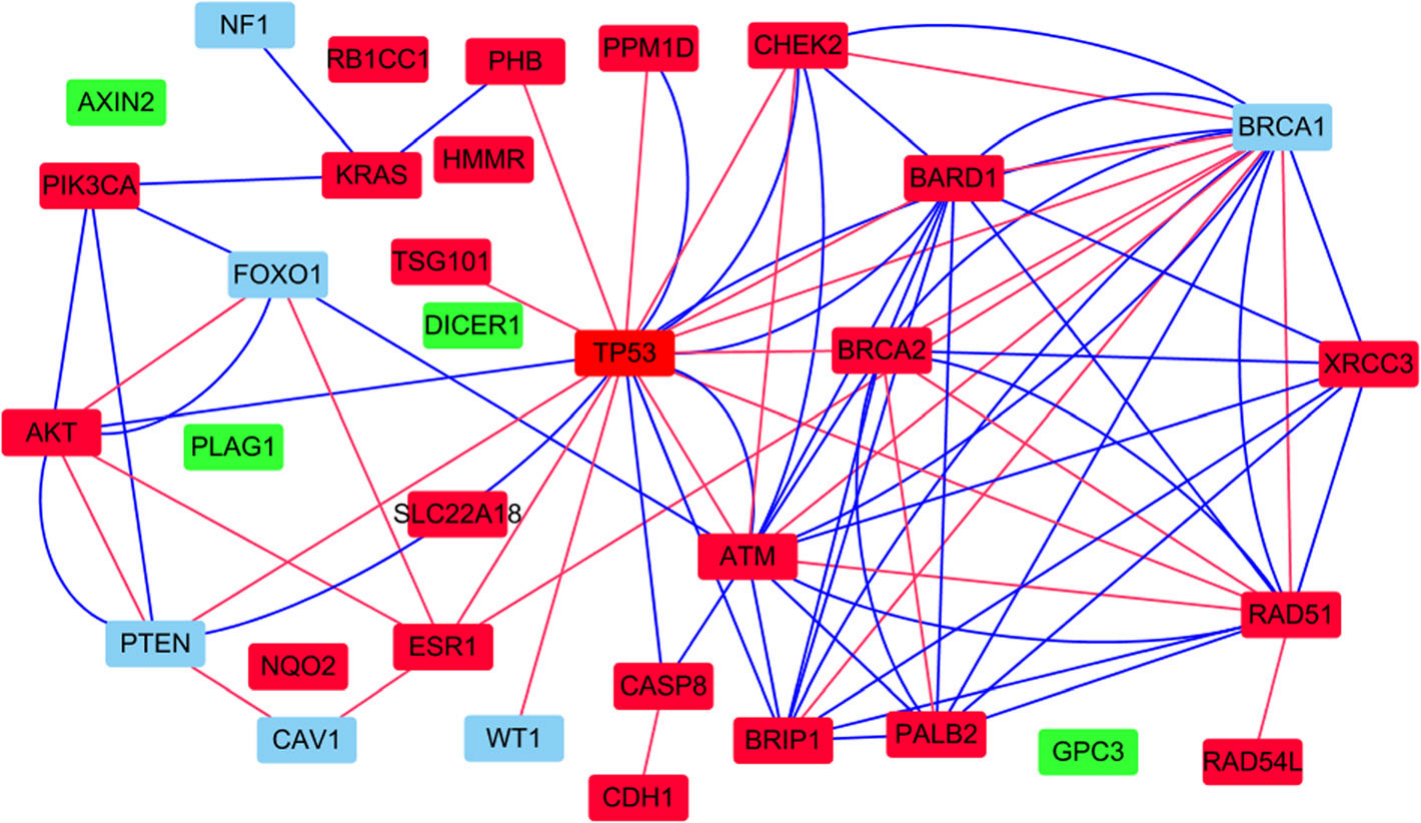
Network represent of breast cancer gene and new top 10 breast cancer gene

To explain the top 10 candidate genes for breast cancer, we analyze them from the perspective of network as Fig. 4. Red nodes and other nodes represent breast cancer genes and 10 candidate genes, respectively in Fig. 4. Besides, red edges and blue edges represent interactions in PPI network and in pathway network, respectively. Obviously, we can see that NF1, BRCA1, FOXO1, PTEN, CAV1 and WT1 are linked with breast cancer genes in PPI network or pathway network. Besides, AXIN2, PLAG1, GPC3, DICER1 are not connected with any breast cancer genes. However, I find AXIN2, PLAG1, GPC3 are association with other cancer diseases. These nodes are marked as green in Fig. 4.

## Conclusion

Due to the lack of labelled genes (test genes), it is a tremendous challenge to identify potential cancer-related genes based on various biological data. In this paper, a TRWR-MB random walk is presented based on multiple types of biological networks. The highlights of our work are to integrate multiple types of biological data and to expand the seed nodes of random walk with restart on quadruple layer heterogeneous network. Experimental results illustrate that TRWR-MB has a satisfactory performance.

Nevertheless, TRWR-MB still has some shortcomings that need to be improved in the future. Firstly, different datasets will have different parameter values. It is a challenge that how to select optimal parameter values. Secondly, compared to various biological data generated by high-throughput biological experimental technique, our integrated biological data is still relatively small. Thirdly, different types of biological data probably contain some noise, which result in a negative effect on constructing quadruple layer heterogeneous network. In conclusion, these shortcomings will encourage us to do continuous researches in the future.

## Supplementary information

**Additional file 1.** It contains manually extracted DOID corresponding to diseases in LncRNADisease database.

**Additional file 2.** It contains all detailed results to describe the effect of parameters.

## Data Availability

The datasets used and/or analyzed during the current study are available from the corresponding author on reasonable request.

## Abbreviations

AUC: A higher value of area under the receiver operating characteristic curve
DO: Disease Ontology
FPR: False positive rate
HPRD: Human Protein Reference Database
LOOCV: Leave-one-out across validation
PPI: Protein-protein interaction
TPR: True positive rate
